# circSPECC1 Promotes Proliferation and Migration of LNCaP Prostate Cancer Cells by Affecting Their Epithelial-Mesenchymal Transition

**DOI:** 10.1155/2023/6956038

**Published:** 2023-03-27

**Authors:** Fangmei Xie, Jian Shen, Zeping Han, Wenfeng Luo, Liyin Liao, Jinhua He

**Affiliations:** ^1^Central Laboratory of Panyu Central Hospital, Guangzhou Panyu Central Hospital, Guangzhou, Guangdong 511400, China; ^2^Laboratory Department of Panyu Central Hospital, Guangzhou, Guangdong 511400, China

## Abstract

**Objective:**

To determine the effects of circSPECC1 (hsa_circ_0000745) on the proliferation and migration of LNCaP prostate cancer cells and to explore the potential molecular mechanism.

**Methods:**

Stable circSPECC1 shRNA-expressing and circSPECC1-overexpressing LNCaP cell lines were constructed, and relative gene expression levels were determined by RT-PCR. MTT and clonogenic assays were used to assess proliferative ability while a scratch test was used to analyze cell migration. Western blotting was used to determine protein expression levels. The effects of circSPECC1 on the proliferation of LNCaP prostate cancer cells were observed in vivo.

**Results:**

circSPECC1 was found to be derived from the SPECC1 (sperm antigen with calponin homology and coiled-coil domains 1) parent gene and to form a loop. Overexpression of circSPECC1 promoted the proliferation and migration of the LNCaP cells, whereas decreased expression of circSPECC1 inhibited these properties. Overexpression of circSPECC1 promoted the expression of MMP-2, MMP-9, VEGF, vimentin, and N-cad but downregulated the expression of E-cad. Decreased expression of circSPECC1 inhibited the expression of MMP-2, MMP-9, VEGF, vimentin, and N-cad but increased the expression of E-cad.

**Conclusion:**

circSPECC1 promotes cell proliferation and migration by affecting the epithelial-mesenchymal transition of LNCaP prostate cancer cells.

## 1. Introduction

Circular RNA (circRNA) is a recently discovered class of endogenous noncoding RNA that forms a special covalently closed circular structure without 5′-3′ polarity or a polyadenylate tail [1]. circRNA is involved in almost all cellular physiological processes, and its abnormal expression is closely related to tumor invasion and metastasis [2].

Prostate cancer is one of the most common malignant tumors in men. Its incidence is lower in China than in Europe and America but is nonetheless increasing year by year, becoming the third most common urogenital cancer in men [3]. Due to the limited methods for early clinical diagnosis, most patients already have middle- or late-stage disease at the initial diagnosis, which leads to poor prognosis and survival [4]. The main reason for the low survival rate is the lack of effective early diagnostic markers and therapeutic targets, and tumor metastasis is the most important independent risk factor for poor prognosis in prostate cancer patients [5]. Studies have found that circRNA plays an important role in tumor genesis and development, but the key circRNA that regulates the progression of prostate cancer and the molecular regulatory mechanism remains unclear.

Previous studies have shown that circSPECC1 is highly expressed in prostate cancer tissues and prostate cancer cell lines [6]. circSPECC1 originates from the SPECC1 (sperm antigen with calponin homology and coiled-coil domains 1) parent gene, which is located at chr17:20107645–20109225^″^. The gene encodes a protein belonging to the cytoplasmic A family, which is localized to the nucleus and highly expressed in the testes and in some cancer cell lines. Chromosomal translocation of this gene is one of the causes of juvenile granulocyte leukemia (http://www.circbase.org/cgi-bin/simplesearch.cgi) [7].

However, the molecular mechanism underlying how circSPECC1 affects the occurrence and development of prostate cancer requires further exploration. In this study, the potential role of circSPECC1 in promoting the occurrence and development of prostate cancer was investigated mainly at the cellular and animal levels. Our results provide a scientific basis for targeted gene therapy and the design of strategies to inhibit the metastasis of prostate cancer.

## 2. Materials and Methods

### 2.1. Cell Culture

The LNCaP cell line was purchased from the Cell Bank of the Chinese Academy of Science (Shanghai, China) and was cultured in Dulbecco's Modified Eagle Medium (C12430-062, Invitrogen, USA) containing 10% fetal bovine serum (C2027050, Invitrogen) at 37°C in a 5% CO_2_ incubator. The cells were digested and cultured with Trypsin-EDTA (0.25%; C25200-072, Invitrogen) every 2–3 days according to cell growth. The cells with a trypan blue rejection rate > 95% in the logarithmic growth stage were selected for experiments.

### 2.2. Construction and Experimental Grouping of Stably Transfected Cell Lines

The design and synthesis of the circSPECC1 siRNA sequence and construction of the lentivirus expression vector were completed by Sangon Biotech (Shanghai, China). The siRNA sequences were as follows: 5′-GGGCCTTTACTCTGCTGGCC-3′ (hsa_circ_0000745) and 5′-GAACTGGGGT GCGTGTGATT T-3′ (negative control). 293T cells were transfected by mixing lentivirus overexpression vector/interference vector and packaging plasmid in 4 : 3. The solution was changed 6 h after transfection, and the lentivirus supernatant was collected and concentrated 48 h later. The LNCaP cells were infected with the lentivirus, and the solution was changed 24 h after infection. The experimental groups comprised a blank control group (LNCaP cells), negative control group (NC, containing empty virus and a negative control sequence), a circSPECC1 shRNA group (stable expression of si-circSPECC1), and a circSPECC1 group (overexpression of circSPECC1).

### 2.3. Real-Time Quantitative Polymerase Chain Reaction

Total RNA was extracted from tissues and cells using an RNAiso Plus kit (9109, TaKaRa). RNA was reverse transcribed into cDNA using the Hifair^®^ II 1st Strand cDNA Synthesis kit (11119ES60, Yeasen). Finally, real-time quantitative polymerase chain reaction (RT-PCR) was performed using AceQ qPCR SYBR Green Master Mix (Q131-02, Vazyme). GAPDH was used as an internal reference. Relative gene expression levels were calculated by the 2^−*ΔΔ*Ct^ method. Gene primer sequences are shown in Table 1.

### 2.4. MTT

The LNCaP cells (10^4^/mL) were plated in 96-well plates at 100 *μ*L/well and cultured for 24, 48, or 72 h. Then, 20 *μ*L of MTT solution (5 mg/mL) was added to each well and incubated at 37°C for 4 h. The supernatant was discarded, and 150 *μ*L DMSO was added to dissolve the crystals. The absorbance value at 490 nm was measured using a Multiskan™ FC microplate photometer (Thermo Fisher Scientific), and the cell proliferation rate was calculated.

### 2.5. Clone Formation

The LNCaP cells (400/well) were plated in 6-well plates and placed in an incubator containing 5% CO_2_ at 37°C for 10–15 days. The culture medium was removed, and the cells were fixed with paraformaldehyde for 5 min, stained with crystal violet, and photographed. The number of clones formed was then calculated.

### 2.6. Scratch Test

The cells in the logarithmic growth phase were cultured in 6-well plates. When the cells reached 100% confluence, they were washed once with phosphate-buffered saline after vertical scratches were made with a pipette tip. Serum-free medium was then added for further culture. After sampling and photographing at 0 and 48 h, ImageJ software was used to calculate the mean distance between the cells.

### 2.7. Western Blotting

Total protein was extracted with RIPA lysis buffer, and its concentration was measured using a BCA Protein Assay Kit (KGP902; KeyGen Biotech, Nanjing, China). Proteins were separated by 10% SDS-PAGE electrophoresis and transferred to a nitrocellulose filter membrane. The membranes were incubated with blocking buffer consisting of 5% nonfat dry milk dissolved in Tris-buffered saline containing 0.1% Tween-20 (TBST), for 60 min at room temperature. After washing the membranes with TBST, they were incubated at 4°C overnight with the following primary antibodies: anti-MMP-2, anti-MMP-9, anti-E-cad, anti-VEGF, anti-Vimentin, anti-N-cad, or anti-GAPDH polyclonal antibodies (diluted 1 : 2000 in blocking solution; Sigma-Aldrich). An enhanced chemiluminescence reagent (KeyGen, Nanjing, China) was added after incubation with HRP-conjugated goat anti-rabbit IgG secondary antibody (1 : 5000 in blocking solution; Sigma-Aldrich) for 1 h at room temperature. A Bio-Rad gel imaging system was used for protein band visualization, GAPDH was used as an internal reference, and the relative protein expression level was calculated by the ratio of the gray value of the target protein to that of GAPDH.

### 2.8. In Vivo Experiments

Twenty 6-week-old male BALB/c nude mice (license no.: SCXK (Guangdong) 2013-002) were subcutaneously injected on the back with a total of 2 × 10^6^ LNCaP cells, NC LNCaP cells, OE-circSPECC1 LNCaP cells, and sh-circSPECC1 LNCaP cells. Every 2 days, the tumor volume was measured using electronic digital calipers and the mice were weighed. After 20 days of continuous monitoring, the mice were sacrificed, the tumor was excised, and the weight of the tumor was recorded.

### 2.9. Statistical Analysis

Statistical analysis was performed using IBM SPSS Statistics 26.0 software. Measurement data are expressed as mean ± standard deviation, one-way ANOVA was used for comparisons between groups, and *P* < 0.05 was considered statistically significant.

## 3. Results

### 3.1. circSPECC1 Is Derived from the SPECC1 Parent Gene and Forms a Loop

**Figure 1:**
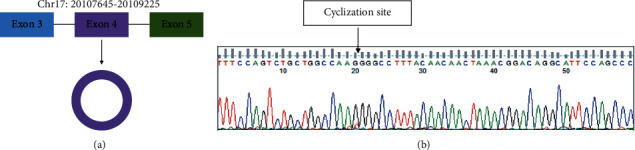
circSPECC1 is derived from the SPECC1 parent gene and forms a loop. (a) circSPECC1 is looped by exon 4 of SPECC1. (b) Sanger sequencing detected the circSPECC1 loop formation.

circSPECC1 (hsa_circ_000745) is derived from the SPECC1 parent gene, located at chr17:20107645–20109225, and consists of exon 4 of SPECC1 (Figure 1(a)). Sanger sequencing results were consistent with the splicing sequence in circBase, which indicated that our circSPECC1 was circRNA (Figure 1(b)).

### 3.2. circSPECC1 Affects the Proliferation and Migration of the LNCaP Cells

**Figure 2:**
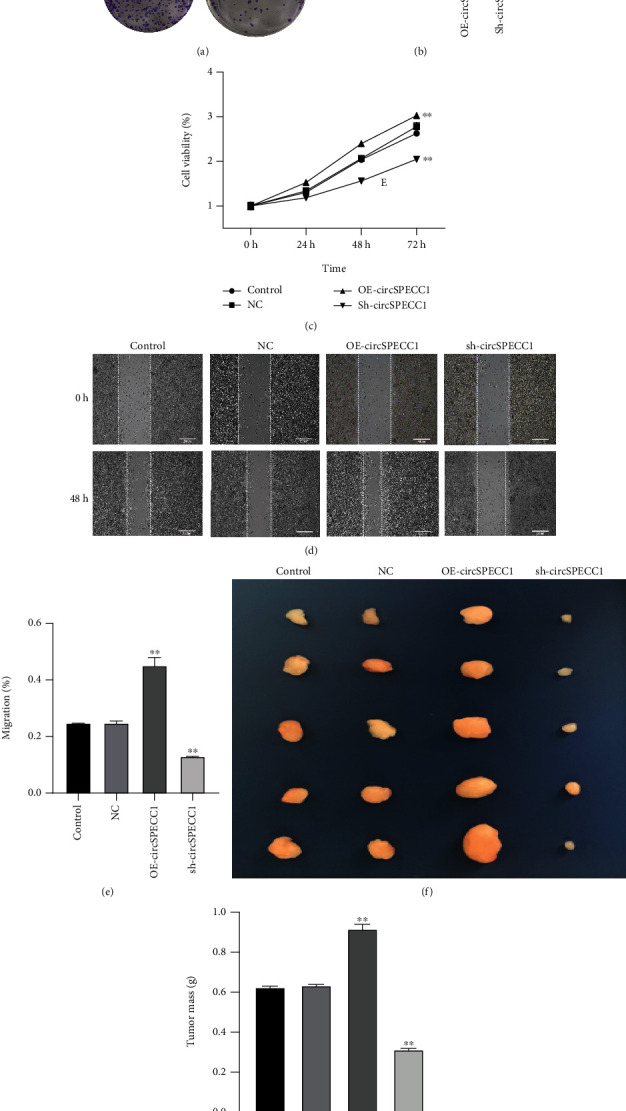
Effects of circSPECC1 on the proliferation and migration of the LNCaP cells. (a) Photographs of cloning experiments. (b) Cell clone counts of the different groups. ^∗∗^*P* < 0.01 compared with the blank control and NC groups. (c) MTT detection of the inhibition rate of cell proliferation. ^∗∗^*P* < 0.01 compared with the blank control and NC groups. (d) Scratch assay to detect cell migration. (e) Comparison of cell mobility in different groups. ^∗∗^*P* < 0.01 compared with the blank control and NC groups. (f) Photograph of tumors. (g) Comparison of tumor weights among the different groups. ^∗∗^*P* < 0.01 compared with the blank control and NC groups.

The LNCaP cells were transfected with circSPECC1 overexpression vector and shRNA-circSPECC1 expression vector for 48 h. The results showed that circSPECC1 promoted cell proliferation, migration, and tumor growth while sh-circSPECC1 inhibited cell proliferation, metastasis, and tumor growth (Figure 2). Thus, circSPECC1 promotes the proliferation and migration of the LNCaP cells. Detailed data were presented in Table 1, Table 2, Table 3, and Table 4 in the Supplementary Material.

### 3.3. circSPECC1 Affects the Epithelial-Mesenchymal Transition of the LNCaP Cells

**Figure 3:**
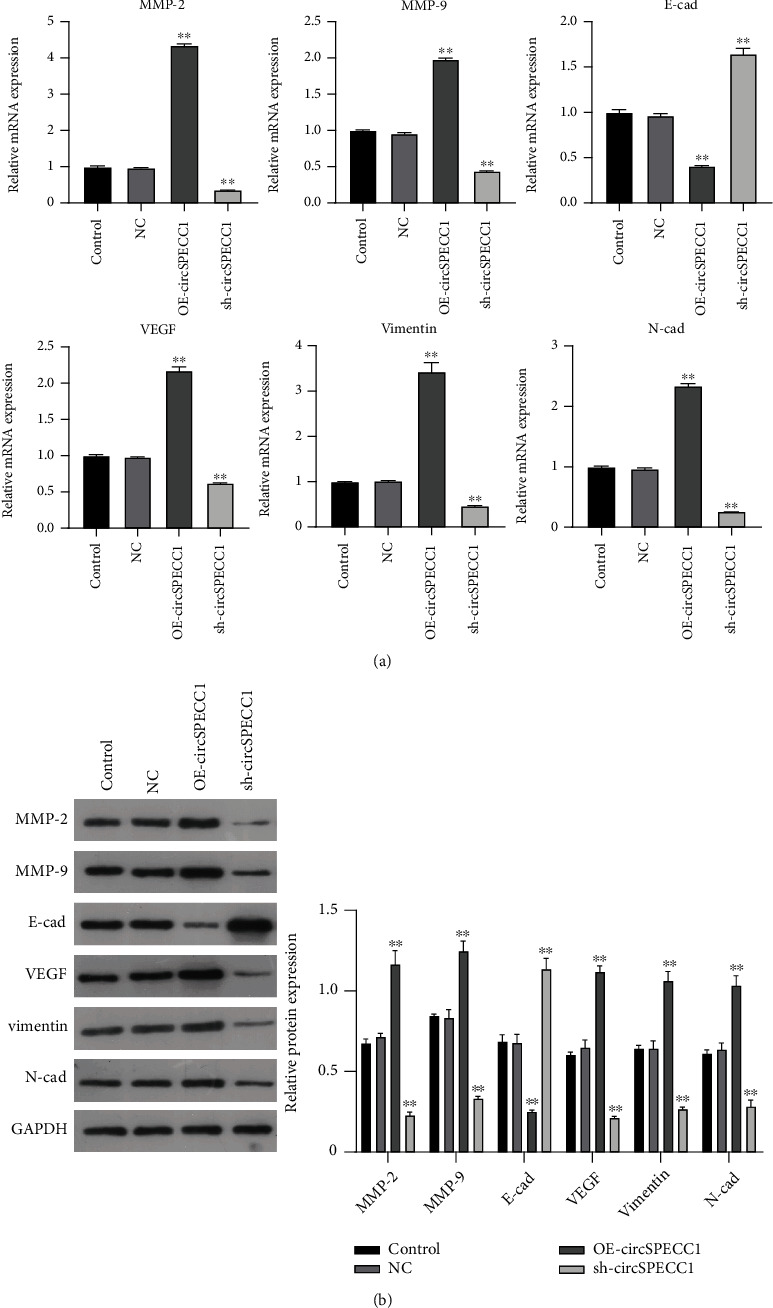
Effects of circSPECC1 on the EMT of the LNCaP cells. (a) RT-PCR detection of the relative expression levels of genes. ^∗∗^*P* < 0.01 compared with the blank control and NC groups. (b) Relative expression levels of proteins detected by western blotting. (A) Representative western blots. (B) Comparison of the relative levels of different proteins. ^∗∗^*P* < 0.01 compared with the blank control and NC groups.

To further explore the potential molecular mechanism underlying the effects of circSPECC1 on the proliferation and migration of the LNCaP cells, we created LNCaP cells with circSPECC1 overexpression and shRNA-circSPECC1 expression. RT-PCR and western blotting were used to determine the expression levels of genes related to the epithelial-mesenchymal transition (EMT). The results showed that overexpression of circSPECC1 promoted the expression of MMP-2, MMP-9, VEGF, vimentin, and N-cad but downregulated the expression of E-cad. Decreased expression of circSPECC1 inhibited the expression of MMP-2, MMP-9, VEGF, vimentin, and N-cad but increased that of E-cad (Figure 3). Thus, circSPECC1 affected the EMT of the LNCaP cells. Detailed data were presented in Table 5 and Table 6 in the Supplementary Material.

## 4. Discussion

circRNAs are effective, specific, and accurate when used as cancer biomarkers [8]. circAGO2, CIRC_0057558, circ_SMARCA5, and circrNA-MyLK are upregulated in prostate cancer tissues and promote the proliferation, migration, and invasion of prostate cancer cells [9–12]. In contrast, circAMOTL1L is downregulated in human prostate cancer, and reduced circAMOTL1L facilitates the migration and invasion of prostate cancer cells by downregulating E-cad and upregulating vimentin, thereby leading to EMT and prostate cancer progression [13]. circRNA_100146 is significantly upregulated in prostate cancer cells, and circRNA_100146 silencing can inhibit their invasion, migration, and proliferation [14]. Overexpression of circRNA_100395 inhibits the proliferation of prostate cancer cells, alters the distribution of cell cycle phases, reduces cell migratory and invasive ability, and inhibits EMT, making it a potential therapeutic target for prostate cancer [15]. In our study, circSPECC1 was derived from the SPECC1 parent gene and formed a loop. Overexpression of circSPECC1 promoted the proliferation and migration of the LNCaP cells, whereas the decreased expression of circSPECC1 had the opposite effect. Thus, our results suggest that circSPECC1 might be a potential target for biological therapies for prostate cancer.

Metastasis of prostate cancer is a multistep, multilink, continuous, and complex process. One important step in the process is the infiltration of tumor cells into the circulatory system through the basement membrane, and EMT plays a key role in this metastasis cascade reaction. EMT causes tumor cells to lose epithelial-like polarity and acquire mesenchymal characteristics, thereby increasing the metastatic and invasive potential of the cells [16]. EMT promotes tumor metastasis by upregulating or downregulating the expression of epithelial markers such as MMP-2, MMP-9, VEGF, vimentin, E-cad, and N-cad [17]. To further explore the potential molecular mechanism underlying the effects of circSPECC1 on the proliferation and migration of the LNCaP cells, we transfected the LNCaP cells for 48 h with a circSPECC1 overexpression vector and shRNA-circSPECC1 expression vector. circSPECC1 overexpression promoted the expression of MMP-2, MMP-9, VEGF, vimentin, and N-cad but downregulated that of E-cad. Decreased expression of circSPECC1 inhibited the expression of MMP-2, MMP-9, VEGF, vimentin, and N-cad but elevated that of E-cad. Thus, circSPECC1 promoted the proliferation and migration of LNCaP prostate cancer cells by affecting EMT. Similarly, hsa_circ_0030586 is significantly overexpressed in prostate cancer cells, and downregulation of hsa_circ_0030586 can reduce the proliferative, migratory, and invasive ability of prostate cancer cells and further inhibit their EMT [18]. In addition, hsa_circ_0001085 may indirectly regulate the PI3K-Akt and TGF-*β* signaling pathways through hsa_miR-196b-5P and the MAPK signaling pathway through hsa_miR-451A, thereby playing a regulatory role in the EMT-induced model of prostate cancer cells [19].

In conclusion, our in vitro and in vivo experiments demonstrated that overexpression of circSPECC1 promoted the proliferation and migration of the LNCaP cells and that decreased expression of circSPECC1 inhibited their proliferation and migration. The likely mechanism is that circSPECC1 promotes the proliferation and migration of LNCaP prostate cancer cells by affecting their EMT. This work provides a scientific basis for targeted gene therapy and the design of strategies to inhibit metastasis in prostate cancer.

## Figures and Tables

**Table 1:** Primer sequences.

Gene	Forward (5′-3′)	Reverse (5′-3′)	Length (bp)
MMP-2	TTTGACGGTAAGGACGGAC	TGGCCTTCTCCCAAGGTCC	107
MMP-9	TCTGCCTGCACCACCGACG	CTGGGTGTAGAGTCTCTCG	114
E-cad	CAACGATAATCCTCCGATCT	ACGGTGACGGTGGCTGTGGA	138
VEGF	CGAGCGGAGCCGCGAGAAGT	ACCCGTCCATGAGCCCGGCT	137
Vimentin	GAAGAGGAAATCCAGGAGCT	TTTCATATTGCTGACGTACGT	118
N-cad	TAATGGAAATCAAGT	ATCCCTCAGGAACTGTCCCA	113
GAPDH	GCTCATTTGCAGGGGGGAG	GTTGGTGGTGCAGGAGGCA	138
hsa-cir-0000745	GCCCAAATGTCGATGGAACA	TTTCTTCCTCTAGATCGGAA	114
U6	CTCGCTTCGGCAGCACA	AACGCTTCACGAATTTGCGT	94

## Data Availability

The raw data supporting the conclusions of this article will be made available by the authors upon request.

## Abbreviations

circRNA: Circular RNA
SPECC1: Sperm antigen with calponin homology and coiled-coil domains 1
MMP-2/MMP-9: Matrix metalloproteinases-2/9
VEGF: Vascular endothelial growth factor
E-cad: E-cadherin
N-cad: N-cadherin
NC: Negative control
GAPDH: Glyceraldehyde-3-phosphate dehydrogenase
RT-PCR: Real-time polymerase chain reaction
BCA: Bicinchoninic acid
MTT: 3-(4,5-Dimethyl-2-thiazolyl)-2,5-diphenyl-2-H-tetrazolium bromide
siRNA: Small interfering RNA
EMT: Epithelial-mesenchymal transition.

## Supplementary Materials

Supplementary Materials Table 1: clone formation. Table 2: cell viability. Table 3: migration (%). Table 4: tumor mass (g). Table 5: relative mRNA expression. Table 6: relative protein expression.
