# Supplementary material for: circSPECC1 Promotes Proliferation and Migration of LNCaP Prostate Cancer Cells by Affecting Their Epithelial-Mesenchymal Transition
Source: J Immunol Res. 2023 Mar 27;2023:6956038. doi: 10.1155/2023/6956038 (PMC10070022; doi:10.1155/2023/6956038)
Supplement: Supplementary Materials — Table 1: clone formation. Table 2: cell viability. Table 3: migration (%). Table 4: tumor mass (g). Table 5: relative mRNA expression. Table 6: relative protein expression. [file 6956038.f1.docx]

Tab 1 Clone formation

| sample | 1 | 2 | 3 | **mean** | **mean±sd** |
| --- | --- | --- | --- | --- | --- |
| Control | 307 | 317 | 309 | 311.00 | 311±5.29 |
| NC | 316 | 302 | 312 | 310.00 | 310±7.21 |
| OE-circ-SPECC1 | 431 | 435 | 429 | 431.67 | 431.67±3.06 |
| sh-circSPECC1 | 90 | 83 | 92 | 88.33 | 88.33±4.73 |

Tab 2 cell viability

| **Time** | **Sample** | **1** | **2** | **3** | **mean** | **mean±sd** |
| --- | --- | --- | --- | --- | --- | --- |
| 0h | Control | 0.304 | 0.345 | 0.343 | 0.34 | 0.33±0.02 |
|  | NC | 0.317 | 0.322 | 0.316 | 0.32 | 0.32±0 |
|  | OE-circSPECC1 | 0.345 | 0.328 | 0.292 | 0.31 | 0.32±0.03 |
|  | sh-circSPECC1 | 0.324 | 0.322 | 0.325 | 0.32 | 0.32±0 |
| 24h | Control | 0.41 | 0.455 | 0.429 | 0.44 | 0.43±0.02 |
|  | NC | 0.435 | 0.413 | 0.431 | 0.42 | 0.43±0.01 |
|  | OE-circSPECC1 | 0.501 | 0.497 | 0.473 | 0.49 | 0.49±0.02 |
|  | sh-circSPECC1 | 0.376 | 0.391 | 0.384 | 0.39 | 0.38±0.01 |
| 48h | Control | 0.665 | 0.682 | 0.676 | 0.68 | 0.67±0.01 |
|  | NC | 0.649 | 0.667 | 0.655 | 0.66 | 0.66±0.01 |
|  | OE-circSPECC1 | 0.784 | 0.767 | 0.763 | 0.77 | 0.77±0.01 |
|  | sh-circSPECC1 | 0.51 | 0.493 | 0.513 | 0.50 | 0.51±0.01 |
| 72h | Control | 0.869 | 0.851 | 0.887 | 0.87 | 0.87±0.02 |
|  | NC | 0.869 | 0.918 | 0.866 | 0.89 | 0.88±0.03 |
|  | OE-circSPECC1 | 0.987 | 0.973 | 0.961 | 0.97 | 0.97±0.01 |
|  | sh-circSPECC1 | 0.651 | 0.673 | 0.664 | 0.67 | 0.66±0.01 |

Tab 3 Migration(%)

| **time** | **sample** | **1** | **2** | **mean** | **mean±sd** |
| --- | --- | --- | --- | --- | --- |
| 0 h | Control | -0.90% | 0.90% | 0.00% | 0±0.01 |
|  | NC | -0.90% | 0.90% | 0.00 | 0±0.01 |
|  | OE-circSPECC1 | -0.54% | 0.54% | 0.00% | 0±0.01 |
|  | sh-circSPECC1 | -1.18% | 1.18% | 0.00% | 0±0.02 |
| 24 h | Control | 25.00% | 24.64% | 24.82% | 0.25±0 |
|  | NC | 25.45% | 24.19% | 24.82% | 0.25±0.01 |
|  | OE-circSPECC1 | 47.28% | 42.93% | 45.11% | 0.45±0.03 |
|  | sh-circSPECC1 | 13.19% | 12.83% | 13.01% | 0.13±0 |

Tab 4 Tumor mass(g)

| **Sample** | **1** | **2** | **3** | **4** | **5** | **mean** | **mean±sd** |
| --- | --- | --- | --- | --- | --- | --- | --- |
| Control | 0.621 | 0.624 | 0.634 | 0.637 | 0.613 | 0.63 | 0.63±0.01 |
| NC | 0.636 | 0.621 | 0.639 | 0.643 | 0.633 | 0.63 | 0.63±0.01 |
| OE-circSPECC1 | 0.973 | 0.916 | 0.913 | 0.901 | 0.884 | 0.91 | 0.93±0.03 |
| sh-circSPECC1 | 0.297 | 0.316 | 0.327 | 0.313 | 0.314 | 0.32 | 0.31±0.02 |

Tab 5 Relative mRNA Expression

| Sample-Gene | Ct1 | Ct2 | Ct3 | 2^-△△Ct^ (mean±sd) |
| --- | --- | --- | --- | --- |
| Control-MMP-2 | 27.31 | 27.27 | 27.39 | 1.00±0.05 |
| NC-MMP-2 | 27.33 | 27.36 | 27.33 | 0.97±0.01 |
| OE-circSPECC1-MMP-2 | 25.16 | 25.21 | 25.2 | 4.35±0.08 |
| sh-circSPECC1-MMP-2 | 28.76 | 28.77 | 28.81 | 0.37±0.01 |
| Control-MMP-9 | 27.31 | 27.27 | 27.3 | 1.00±0.02 |
| NC-MMP-9 | 27.33 | 27.36 | 27.31 | 0.96±0.02 |
| OE-circSPECC1-MMP-9 | 26.31 | 26.27 | 26.3 | 1.98±0.03 |
| sh-circSPECC1-MMP-9 | 28.46 | 28.51 | 28.49 | 0.44±0.01 |
| Control-Ecad | 28.03 | 27.97 | 28.06 | 1.00±0.04 |
| NC-Ecad | 28.04 | 28.09 | 28.02 | 0.96±0.02 |
| OE-circSPECC1-Ecad | 29.26 | 29.31 | 29.33 | 0.41±0.01 |
| sh-circSPECC1-Ecad | 27.25 | 27.36 | 27.33 | 1.65±0.07 |
| Control-VEGF | 26.97 | 26.89 | 26.91 | 1.00±0.03 |
| NC-VEGF | 26.94 | 26.93 | 26.91 | 0.98±0.01 |
| OE-circSPECC1-VEGF | 25.84 | 25.76 | 25.77 | 2.17±0.06 |
| sh-circSPECC1-VEGF | 27.61 | 27.67 | 27.57 | 0.62±0.02 |
| Control-Vimentin | 28.13 | 28.06 | 28.09 | 1.00±0.03 |
| NC-Vimentin | 28.02 | 28.07 | 28.03 | 1.02±0.02 |
| OE-circSPECC1-Vimentin | 26.21 | 26.33 | 26.37 | 3.43±0.20 |
| sh-circSPECC1-Vimentin | 29.13 | 29.21 | 29.22 | 0.47±0.02 |
| Control-Ncad | 26.67 | 26.71 | 26.73 | 1.00±0.02 |
| NC-Ncad | 26.69 | 26.72 | 26.77 | 0.97±0.03 |
| OE-circSPECC1-Ncad | 25.43 | 25.49 | 25.47 | 2.34±0.05 |
| sh-circSPECC1-Ncad | 28.61 | 28.63 | 28.67 | 0.27±0.01 |
| Control-GAPDH | 8.73 | 8.77 | 8.78 | / |
| NC-GAPDH | 8.73 | 8.7 | 8.78 | / |
| OE-circSPECC1-GAPDH | 8.75 | 8.79 | 8.7 | / |
| sh-circSPECC1-GAPDH | 8.8 | 8.75 | 8.77 | / |

Tab 6 Relative Protein Expression

|  | MMP-2 (mean±sd) | MMP-9 (mean±sd) | Ecad (mean±sd) | VEGF (mean±sd) | Vimentin (mean±sd) | N-cad (mean±sd) |
| --- | --- | --- | --- | --- | --- | --- |
| Control | 0.67±0.03 | 0.84±0.01 | 0.68±0.04 | 0.6±0.02 | 0.64±0.02 | 0.61±0.02 |
| NC | 0.71±0.02 | 0.83±0.05 | 0.68±0.05 | 0.65±0.05 | 0.64±0.05 | 0.63±0.04 |
| OE-circSPECC1 | 1.16±0.09 | 1.25±0.06 | 0.25±0.01 | 1.12±0.04 | 1.06±0.06 | 1.03±0.06 |
| sh-circSPECC1 | 0.23±0.02 | 0.33±0.01 | 1.13±0.07 | 0.21±0.01 | 0.27±0.01 | 0.28±0.04 |
